# Fatal Infection with Dengue Virus and Avian Orthoavulavirus 1 in Traveler Returning from Saudi Arabia to France, 2025

**DOI:** 10.3201/eid3210.260736

**Published:** 2026-10

**Authors:** Maud Salmona, Salomé Schlupmann, Morgane Mailhe, Dorothée Chopin, Quentin Richier, Karine Lacombe, Lila Bouadma, Jean-Francois Timsit, Vincent Bondet, Quentin Le Hingrat, Jérôme Le Goff, Jamie Sugrue, Darragh Duffy, Olivier Schwartz, Michael White, Valérie Paradis, Franck Verdonk, François-Xavier Lescure, Marie Jaspard

**Affiliations:** Hôpital Saint-Louis, Paris, France (M. Salmona, J. Le Goff); Hôpital Saint-Antoine, Paris (S. Schlupmann, D. Chopin, Q. Richier, K. Lacombe, M. Jaspard); Hôpital Bichat Claude-Bernard, Paris (M. Mailhe, F.-X. Lescure); Sorbonne Université, Paris (Q. Richier, K. Lacombe, F. Verdonk); Assistance Publique—Hôpitaux de Paris, Paris (L. Bouadma, J.-F. Timsit, Q. Le Hingrat, V. Paradis, F.-X. Lescure); IAME, INSERM, Université Paris Cité, Paris (L. Bouadma, J.-F. Timsit, F.-X. Lescure); Université Paris Diderot, Paris (J.-F. Timsit, F.-X. Lescure); Institut Pasteur, Paris (V. Bondet, J. Sugrue, D. Duffy, O. Schwartz, M. White)

**Keywords:** dengue, avian orthoavulavirus 1, viruses, paramyxovirus, pigeon paramyxovirus 1, zoonoses, vector-borne infections, travel, Saudi Arabia, France

## Abstract

A previously healthy 26-year-old man died of hemorrhagic fever with fulminant hepatitis and shock 36 hours after returning to France from Mecca, Saudi Arabia. Postmortem analyses detected low-level dengue virus RNA and positive NS1 antigen along with unexpected extremely high-titer disseminated velogenic avian orthoavulavirus 1 infection with elevated systemic cytokine responses.

A previously healthy 26-year-old man sought care in Paris, France, immediately after returning from Mecca, Saudi Arabia, in January 2025. He was experiencing fever, myalgia, headache, abdominal pain, and diarrhea that began 4 days earlier. He reported no specific exposure during his trip, including no contact with animals, contaminated food or water, or insect bites. He had no history of immunosuppression or repeated infection. No family members traveling with him experienced similar symptoms. We report the patient’s clinical course and laboratory findings.

## The Study

At admission to the intensive care unit in Saint-Antoine hospital (Paris, France), the patient had fever (40°C), tachypnoea (45 breaths/min, SpO_2_ 95% on room air), poor tissue perfusion, and oliguria. Results of cardiopulmonary, abdominal, and neurologic examination were unremarkable. Conjunctival hemorrhage and hematuria were present at hospital admission. Despite early fluid resuscitation and empirical antimicrobial treatment, his condition rapidly deteriorated. He experienced multiple organ dysfunction syndrome including hypotension, purpuric livedo, anuria, and acute kidney failure (creatinine 600 μmol/L), elevated level of lactate (4.4 mmol/L), major hepatic dysfunction (alanine aminotransferase and aspartate aminotransferase levels were >100 times the upper limit of normal and factor V was at 51%), thrombocythemia (29 × 10^9^/L), decreased hematocrit (39%), decreased prothrombin time (52%), and disseminated intravascular coagulation. Inflammatory markers were elevated (C-reactive protein 320 mg/L; procalcitonine 136 µg/L). We could not perform bacterial analysis because the patient was isolated for initial suspicion of highly infectious pathogens. Body computed tomography results were unremarkable. We applied resuscitation measures and escalated antimicrobial therapy.

Etiologic assessment included PCR testing for malaria, SARS-CoV-2, influenza, cytomegalovirus, Ebola virus, Zika virus, chikungunya virus, and serology for HIV and for hepatitis A, B, C, and E; all results were negative. At that time, the patient’s blood tested negative by PCR and IgM and IgG serology for dengue virus (DENV). The patient’s condition continued to deteriorate; he experienced refractory shock, fulminant hepatitis, and disseminated intravascular coagulation. He died 36 hours after hospital admission.

Postmortem laboratory results revealed a low dengue viral load in urine with quantitative reverse transcription PCR results at a cycle threshold (Ct) of 39 from a pan-dengue duo test and Ct of 40 from a dengue-1–specific test; both had a robust fluorescence curve. Serum was positive for nonstructural 1 glycoprotein antigen (positivity ratio 2.5) by VIDAS Dengue NS1 (bioMérieux, https://www.biomerieux.com). Postmortem liver biopsy demonstrated normal architecture and extensive parenchymal necrosis without any viral inclusions (Figure 1). 

**Figure 1:**
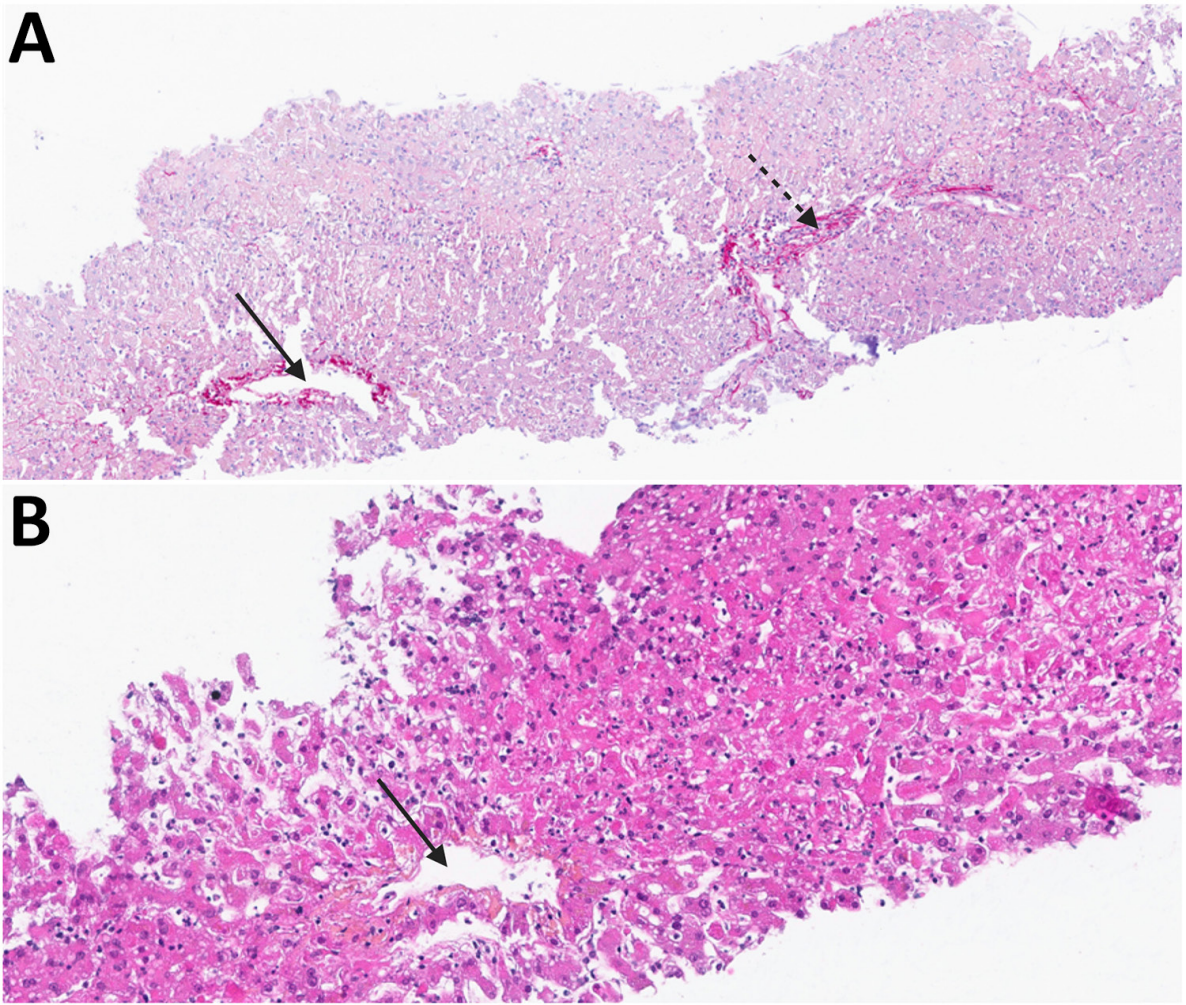
Liver biopsy images from study of a fatal infection with dengue virus and avian orthoavulavirus 1 in traveler from Saudi Arabia, 2025. A) Sirius red stain showing normal architecture without any fibrosis. Solid arrow indicates centrilobular vein; dashed arrow indicates portal tract. Original magnification ×100. B) Hematoxylin and eosin stain showing extensive parenchymal necrosis; all hepatocytes of the lobule are necrotic. Lobules contain few inflammatory cells. Solid arrow indicates centrilobular vein. Original magnification ×200.

We further analyzed plasma and liver biopsy samples using metagenomic shotgun next-generation sequencing (mNGS). mNGS detected avian orthoavulavirus 1 (AOAV-1), the virus historically associated with Newcastle disease, at extremely high levels; 191,718 reads per million in liver tissue, and 244,781 reads per million in plasma. We obtained complete coverage of all AOAV-1 coding sequences (GenBank accession no. PX575854). Phylogenetic analysis classified the strain as AOAV-1 clade II, genotype VI, subgenotype VI.2.1.2 (Figure 2), a pigeon-adapted lineage historically termed pigeon paramyxovirus type 1, with a velogenic fusion protein cleavage site motif (RRRKRF) (*1*). Velogenic strains are highly virulent AOAV-1 variants defined by a polybasic fusion-protein cleavage site, which enables ubiquitous protease activation and systemic multiorgan tissue tropism, in contrast to low-virulence strains, which carry a monobasic cleavage site and remain confined to the respiratory and enteric tract (*1*).

**Figure 2:**
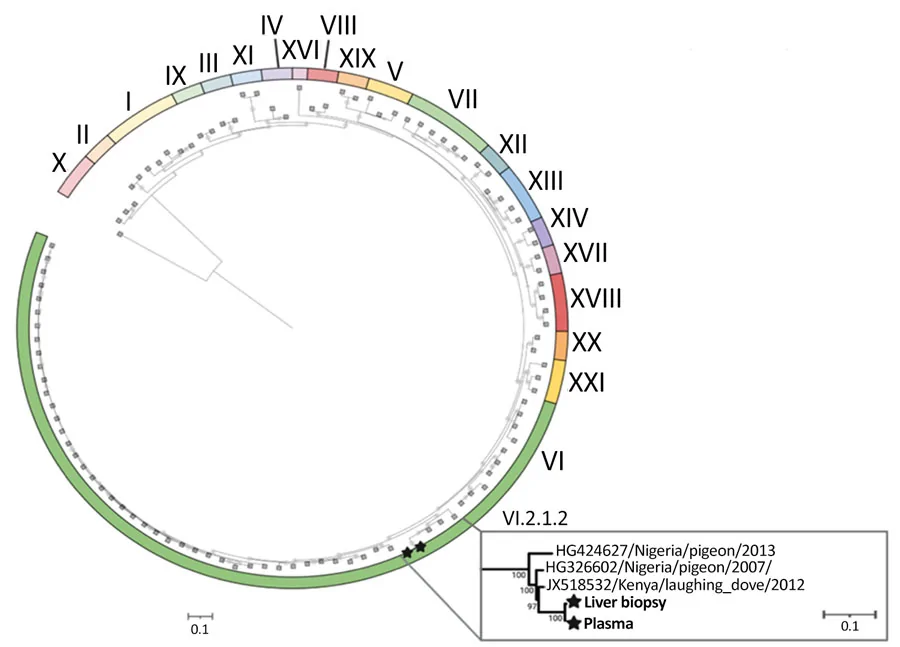
Maximum-likelihood phylogenetic tree of APMV-1 from study of a fatal infection with dengue virus and avian orthoavulavirus 1 in traveler from Saudi Arabia, 2025. The tree was inferred from 120 complete APMV-1 clade II genomes retrieved from GenBank. Black stars indicate sequences obtained from the patient’s liver biopsy and plasma samples, which cluster within subgenotype VI.2.1.2 (inset). Scale bar indicates nucleotide substitutions per site. APMV-1, avian paramyxovirus type-1.

We confirmed AOAV-1 infection by specific real-time PCR detection on postmortem liver biopsy (Ct 17.23) and plasma (Ct 15.74) samples, as well as on premortem urine (Ct 18.23), cerebrospinal fluid (Ct 35.9), and sputum (Ct 18.5) specimens, confirming a disseminated infection. In addition, analysis of the plasma type I interferon (IFN) response by single molecule array digital-ELISA using an in-house Simoa bead-based assay on the HD-X platform (Quanterix, https://www.quanterix.com) showed extremely high levels of IFN-α (>10^4^ pg/mL) (Figure 3, panel A) and IFN-β (>10^4^ pg/mL, data not shown), as well as extremely high levels of IFN-γ, C-X-C motif chemokine ligand 10 and 11, interleukin 6, and tumor necrosis factor-α measured by Luminex (R&D Systems, Inc., https://www.rndsystems.com) (Figure 3, panels B–F).

**Figure 3:**
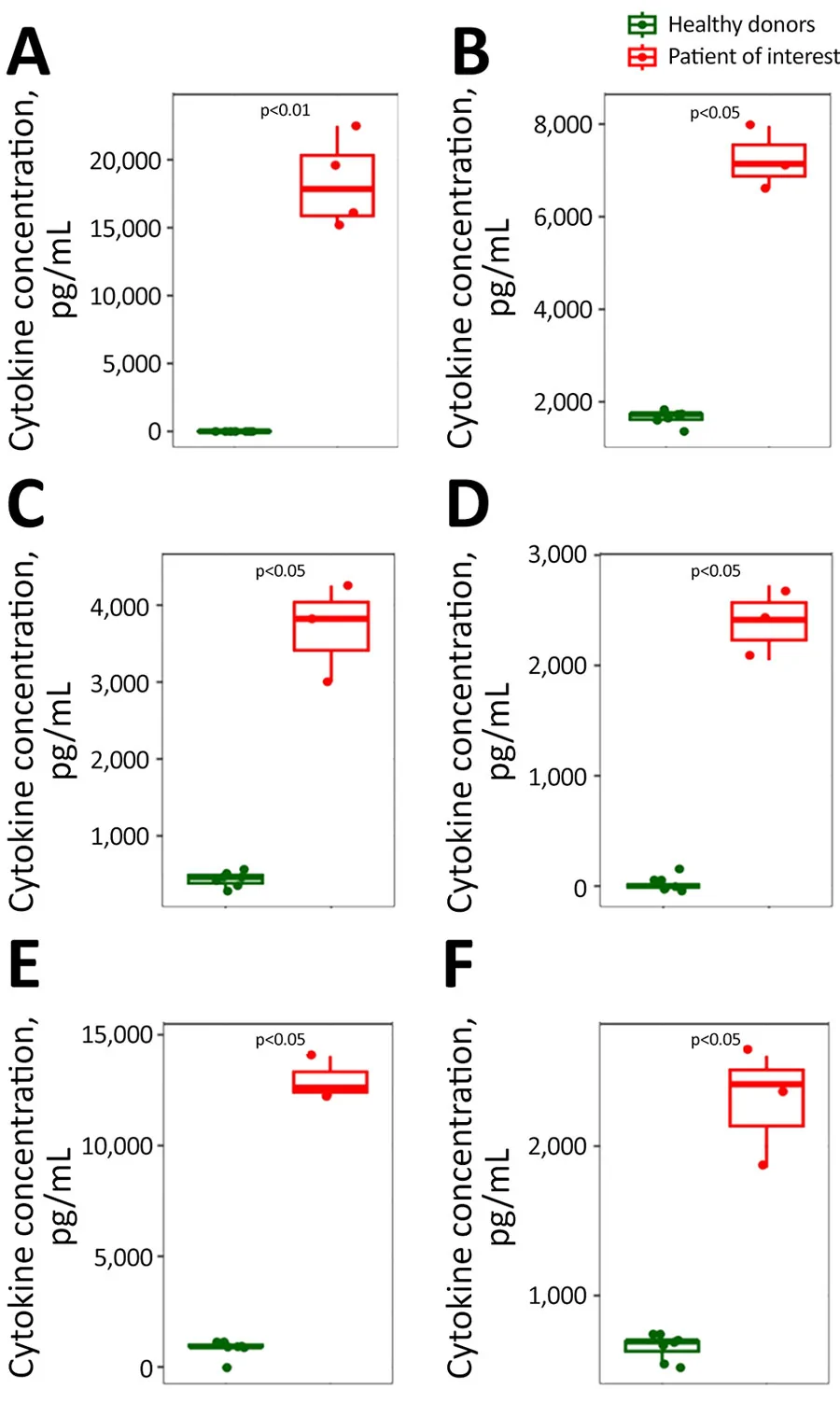
Cytokine concentrations from study of a fatal infection with dengue virus and avian orthoavulavirus 1 in traveler from Saudi Arabia, 2025. A) IFN-α. B) IFN-γ. C) IFN-induced CXCL10. D) IFN-induced CXCL11. E) Pro-inflammatory IL-6. F) Pro-inflammatory TNF-α. We measured concentrations in blood samples from the study patient compared with those from healthy donors. Boxes indicate 95% CI; horizontal bars within boxes indicate median; whiskers indicate ranges; dots indicate individual patient samples. We measured concentrations for interferon-α using single molecule array digital-ELISA using an in-house Simoa bead-based assay on the HD-X platform (Quanterix, https://www.quanterix.com) and multiplexed Luminex assay (R&D Systems, Inc., https://www.rndsystems.com) for other cytokines. We determined p values by Mann-Whitney test. CXCL, C-X-C motif chemokine ligand; IFN, interferon; IL, interleukin; TNF, tumor necrosis factor-α.

## Conclusions

We describe a fulminant febrile illness associated with 2 unusual virologic findings: low-level evidence of DENV infection and high-titer disseminated AOAV-1. We could not establish the relative contribution of each virus to the fatal syndrome with certainty. However, the high AOAV-1 loads detected in plasma and multiple tissues and body fluids provide strong evidence of disseminated infection and a major role for AOAV-1 in the clinical manifestation of the infection.

Severe forms of dengue occur in ≈5% of cases (*2*). Because this patient had no history of dengue infection and no detected DENV IgG, his illness was consistent with primary dengue fever, which although typically milder than subsequent infections, can also lead to severe disease (*2*). Initial blood PCR and serology were negative for DENV, as was the mNGS performed in the liver biopsy. We diagnosed dengue on the basis of DENV-1–specific and pan-dengue PCR assay on urine and the detection of NS1 antigen in blood. 

AOAV-1 infection was detected after patient’s death and was completely unexpected. The AOAV-1 strain genotype VI.2 is a lineage maintained primarily in a pigeon/columbiform reservoir (*3*–*5*). Genotype VI.2.1 has been reported circulating in backyard pigeons in eastern Saudi Arabia (*6*); pigeons are numerous around the Grand Mosque in Mecca, where the patient stayed for 2 weeks. In immunocompetent hosts, human AOAV-1 infection is typically an uncomplicated, self-limited, localized illness, most often manifesting as conjunctivitis with or without mild systemic symptoms; it occurs predominantly in poultry workers exposed to infected flocks (*7*). Infection is more severe in immunosuppressed patients, in whom it causes pulmonary disease with symptoms ranging from upper respiratory tract disorder to fatal respiratory failure with large infiltrative lesion of the lung, and neurologic disorders such as seizure and encephalitis (*8*–*10*). No cases of hepatitis have been reported in humans, although AOAV-1 has been detected in liver biopsy samples (*10*). However, hepatitis caused by AOAV-1 remains plausible; velogenic strains have a broad tropism in birds (*11*). 

Since the advent of mNGS, the number of severe human cases reported in the literature has increased, suggesting that human AOAV-1 infections have previously been underestimated. As of March 2026, a total of 7 severe human AOAV-1 cases had been reported globally: 4 pneumonia and 3 encephalitis cases (*8*–*10*,*12*–*15*). Four patients were immunosuppressed (*8*–*10*,*13*). Disseminated infection was confirmed in 3 cases. Proximity to pigeons was documented in 2 cases (*12*,*14*). Six cases resulted in death. Our case-patient, like 3 others (*12*,*14*,*15*), showed no evidence of immunosuppression related to a known disease or treatment. However, we cannot exclude potential inborn genetic errors affecting immune response. Indeed, we observed a cytokine storm suggestive of an inadequate immune response, which raised questions about possible genetic immune deficiencies. As in our case, all patients experiencing severe infection harbored a velogenic AOAV-1 strain, which is characterized by a multibasic cleavage site in the fusion protein enabling extensive tissue tropism and widespread dissemination.

In summary, the fatal combination of DENV and AOAV-1 infections most likely reflected a multifactorial process. The extremely high, disseminated AOAV-1 loads and the associated extreme type I/III interferon and pro-inflammatory cytokine response are the best-documented and most plausible contributors to the fulminant clinical course. A causal contribution from DENV, despite its low viral load, cannot be excluded. However, our case, together with previous reports, highlights the potential for severe disseminated disease after infection with velogenic AOAV-1 strains. Our findings underscore the value of considering AOAV-1 infection and of applying mNGS in unexplained severe febrile illness after travel.
